# Investigating the role of health care at birth on inequalities in neonatal survival: evidence from Bangladesh

**DOI:** 10.1186/1475-9276-12-17

**Published:** 2013-03-06

**Authors:** Sarah E Neal, Zoë Matthews

**Affiliations:** 1 Centre for Global Health, Population, Poverty and Policy, Department of Social Science, University of Southampton, University Rd, Southampton, Hampshire, SO17 1BJ, United Kingdom

**Keywords:** Neonatal, Inequalities, Developing countries, Maternal health services, Poverty, Access to health care

## Abstract

**Introduction:**

In countries such as Bangladesh many women may only seek skilled care at birth when complications become evident. This often results in higher neonatal mortality for women who give birth in institutions than for those that give birth at home. However, we hypothesise that this apparent excess mortality is concentrated among less advantaged women. The aim of this paper is to examine the association between place of birth and neonatal mortality in Bangladesh, and how this varies by socio-economic status.

**Methodology:**

The study is based on pooled data from four Bangladesh Demographic and Household Surveys, and uses descriptive analysis and binomial multivariate logistic regression. It uses regression models stratified for place of delivery to examine the impact of socio-economic status and place of residence on neonatal mortality.

**Results:**

Poor women from rural areas and those with no education who gave birth in institutions had much worse outcomes than those who gave birth at home. There is no difference for more wealthy women. There is a much stronger socio-economic gradient in neonatal mortality for women who gave birth in institutions than those who delivered at home.

**Conclusion:**

In Bangladesh babies from lower socio-economic groups and particularly those in rural areas have very poor outcomes if born in a facility. This suggests poorer, rural and less educated women are failing to obtain the timely access to quality maternal health care services needed to improve newborn outcomes.

## Introduction

It is well known that the newborns of women with higher socio-economic status have improved rates of neonatal survival compared with their lower status counterparts [1]. However, as institutional births become more common it could be argued that differing patterns of health service use between rich and poor women will exacerbate and widen the mortality gap. Poorer women are less likely to go to a facility to give birth, using services only as a last resort once complications arise. These differences between rich and poor could also be mirrored by differences between educated and non-educated women, and across other socio-economic divides.

The aim of this paper is to examine how neonatal outcomes from institutional deliveries vary for women from different socio-economic groups, and ascertain whether wealth or education makes a greater contribution to mortality gaps for institutional than home births. Our approach is to use both descriptive statistics and stratified multivariate logistic regression models to examine how socio-economic status is associated with neonatal outcomes for women giving birth at home and in institutions.

### Inequities in neonatal mortality and use of health services: an explanatory model

Socio-economic inequalities in neonatal mortality are seen throughout the world [1], and indeed even in developed countries such as the UK newborns born into the poorest families are more likely to die than those from more wealthy backgrounds [2]. Socio-economic status is likely to influence neonatal survival through a range of proximate determinants such as maternal nutrition, birth spacing and family practices. However, there is evidence to suggest that at a national level a much greater proportion of the variance in neonatal mortality can be explained by access to health care rather than contextual factors such as income, female literacy, water and sanitation [3]. This may suggest that for neonates the most important pathway through which socio-economic inequalities affect outcomes is differential access to, and use of health care by rich and poor, or by educated and uneducated.

Care at birth is seen as the cornerstone of efforts to reduce neonatal mortality. However, in countries such as Bangladesh, where skilled care at birth is rare, those who receive it are much more likely to have experienced complications that are strongly associated with the probability of a neonatal death. This results in higher neonatal mortality for women giving birth in institutions in a range of settings [4], but this apparent disadvantage may mask importance differences in groups of women that access services. Studies have consistently demonstrated that wealthy or more educated women are much more likely to deliver in an institution or with skilled care [5-7], which may suggest that a larger proportion of these urban, richer or more educated women will have planned their birth at an institution rather than waiting for a complication to arise.

**Figure 1:**
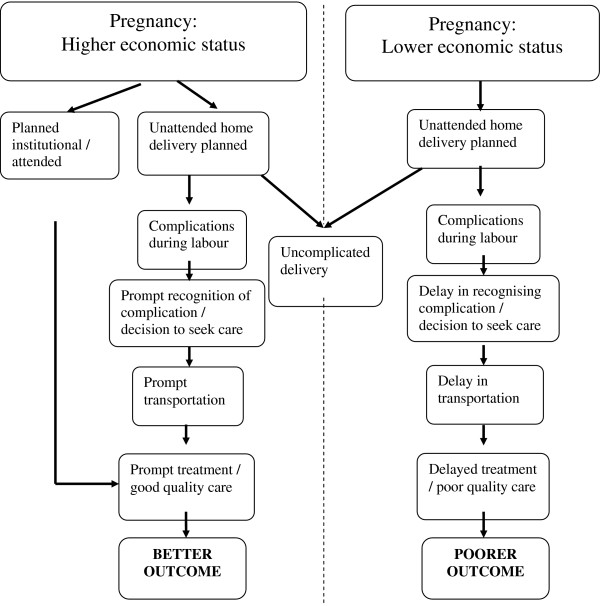
Trajectories to skilled care: a framework to explain inequalities in institutional outcomes for neonates in countries with low levels of skilled attendance.

Even among those who access care only once complications arise, there is evidence that patterns of utilisation are different among the rich and poor. The Bangladesh Maternal Health Survey conducted in 2001 [8] shows that poorer and less educated women experienced longer delays in recognition and decision to seek treatment for both life-threatening and non-life threatening complications. They also experienced longer journey times once the decision to seek care had been made. Further inequities may result from differential quality of care among rich and poor, even if they used the same institution [9]. Figure 1 presents a simplified explanatory framework that demonstrates how the trajectories through which richer and poorer women receive care at birth may influence the outcomes for newborns.

#### Neonatal health within the context of Bangladesh

**Figure 2:**
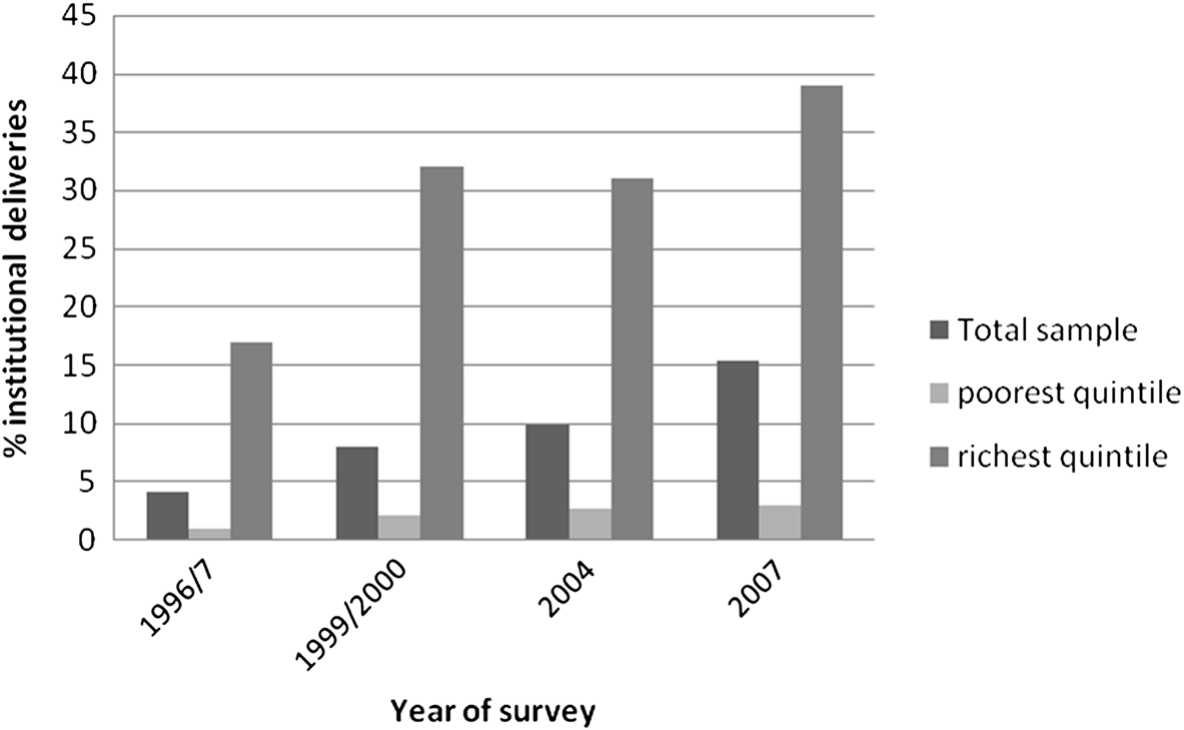
Increase in percentage of institutional births 1996/7 – 2007 by whole sample and richest and poorest quintile.

Neonatal mortality has fallen in Bangladesh over the last four decades from an estimated 74 per 1000 in 1975 [10] to 27 per 1000 in 2009 [11]. Much of this decline is thought to be as a result of efforts to reduce neonatal tetanus through maternal immunisation [12]. While the reduction is significant, as in many countries neonatal mortality has tended to fall less sharply than deaths in older children. As a result, neonatal mortality now makes up nearly half of all deaths in Bangladesh in children under the age of five, and about two-thirds of infant deaths. While Bangladesh has made significant progress in some areas of reproductive health such as uptake of family planning methods, indicators of safe motherhood have not markedly improved. Most recent estimates suggest that 27% give birth with a skilled attendant [11] and while uptake of institutional care at delivery at birth has increased, progress is slow, as illustrated by figures from the four surveys included in our study (see Figure 2). Uptake is particularly low for the poorest quintile where little progress has been made.

## Methods

Our analysis is based on data collected from four Demographic and Household surveys (DHS) collected in 1996/7, 1999/2000, 2004 and 2007 in Bangladesh which were pooled to produce a large sample size. These are nationally representative household surveys providing a combined sample size of 26,079 children born to 19,548 mothers within the previous five years prior to the three surveys. Full birth histories were collected from women aged between 15 and 49 years in sampled households, and data are comparable over time.

In our analysis we use asset quintiles disaggregated for urban/rural residence as a proxy for wealth. DHS normally creates five asset quintiles for the whole population whether rural or urban. The disadvantage of this is that some of the index components do not effectively capture the different patterns of rural and urban asset ownership, and Houweling *et al.*[13] recommends that efforts are made to more accurately quantify assets pertinent to rural communities, *e.g.* land ownership and livestock. In response to this issue, asset scores and quintiles were recalculated separately for urban and rural populations. The same methodology and variables were used as in the original DHS: a principal component analysis (PCA) was run on the selected variables for urban and rural dwellers separately, and an individual score estimated for each case, which became the asset score. Quintiles were then calculated separately for urban and rural dwellers, creating a total of 10 categories. However sample sizes for institutional deliveries among the poorer quintiles were very low, so quintiles 1–4 were amalgamated^a^ to create four groupings: Rural quintile 5 (richest) and rural quintiles 1–4, urban quintile 5 (richest) and urban quintile 1–4.

Initially crosstabulations and Pearson’s Chi Squared tests were used to examine the associations between NMR and urban/rural-quintiles and place of birth. While such descriptive approaches are valuable in identifying general patterns of socio-economic inequalities, interpretation is difficult as many of the factors affecting neonatal mortality are closely correlated. Binomial multivariate logistic regression was therefore carried out to predict neonatal mortality in each of the categories net of other effects. We used a series of six stratified models to examine the impact of wealth/urban rural residence and education on neonatal outcomes at home or in institutions. The models also controlled for year of survey and a number of factors known to be associated with neonatal mortality: sex, multiple birth, birth order, parity and antenatal care. Several other factors were originally included (*e.g.* maternal age) but were removed as they were not significant in any of the models and did not change the coefficient of interest. The models with level of education as explanatory variables also had urban/rural as a control variable.

Our outcome is neonatal mortality, which is defined as death of a live-born infant before the 28^th^ day of life. Originally it had been hoped to also use early neonatal mortality (as a more direct outcome of delivery care) as an outcome variable, but small sample sizes and possible data problems made this impossible. As there may be significant correlations for many unobservable characteristics between births to the same mother the data are clustered using the mother’s identity number in order to adjust the standard errors. We did not disaggregate for the different types of facility (*e.g.* public, private, NGO) due to small sample size, and exploratory analysis suggested it made little difference.

## Results

The overall NMR for the sample within our appended four dataset was 41 per 1000 live births. Around 17% of the (weighted) sample were from urban areas, with 62% in the rural quintile 1–4 grouping. The percentage of women who gave birth in an institution is presented in Table 1 disaggregated by urban/rural wealth and mother’s education. It clearly demonstrates a strong socio-economic gradient, with educated women more likely to give birth in an institution. There is also a clear difference between urban and rural residents, with both rich and poor women in urban areas more likely to give birth in institutions than their rural counterparts: indeed poorer urban women are slightly more likely to have an institutional birth than rural wealthy women.

**Table 1:** Coverage for home and institutional deliveries by socio-economic grouping (numbers in brackets)

	**Urban quintiles 1-4**	**Urban quintiles 5**	**Rural quintiles 1-4**	**Rural quintiles 5**	**Total sample**
**Home deliveries**	83% (2840)	41% (391)	96% (16278)	86% (3630)	89% (23149)
**Institutional deliveries**	17% (586)	59% (561)	4% (642)	14% (595)	11% (2803)
	**No education**	**Primary education**	**Secondary education**	**Further education**	**Total sample**
**Home deliveries**	98% (10816)	94% (7268)	82% (4998)	44% (474)	89% (23149)
**Institutional deliveries**	2% (259)	6% (444)	18% (1093)	56% (594)	11% (2803)

### Descriptive statistics

**Figure 3:**
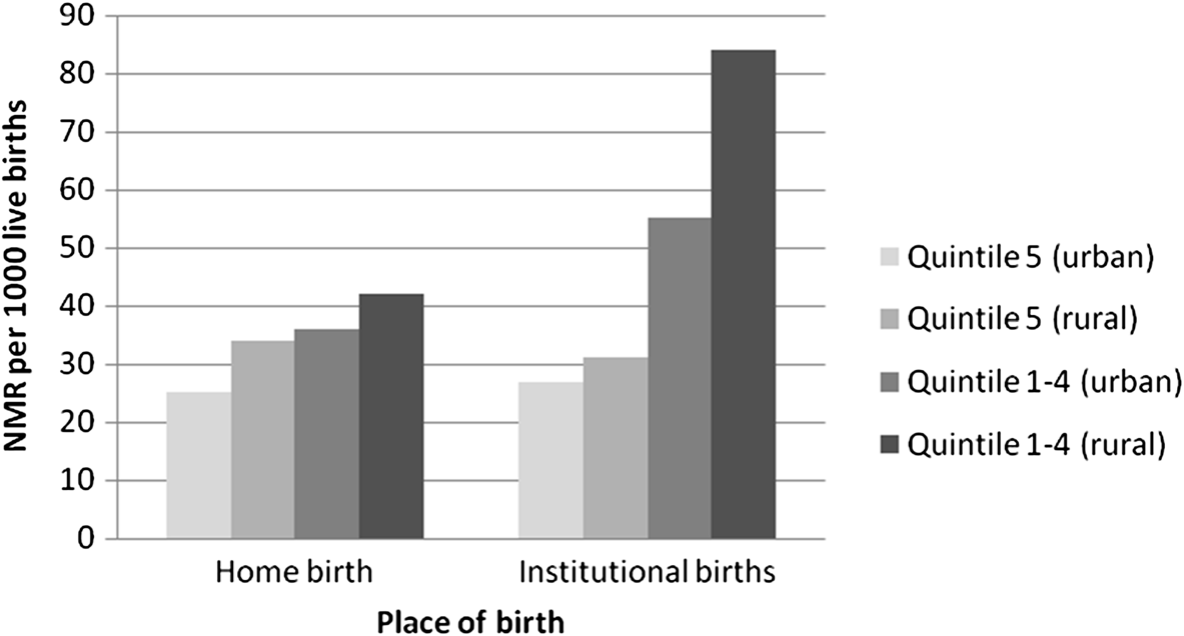
Neonatal mortality by wealth quintile and place of birth.

Table 2 and Figure 3 show NMR disaggregated by urban/rural/wealth and place of birth. When wealth quintile 5 (richest) is considered, there is little difference in NMR between those who gave birth at home or in an institution for both urban and rural areas. For wealth quintile 1–4 (all but richest) that picture is different: there is a marked increase in NMR for urban dwellers giving birth in institutions, but the greatest difference is among rural dwellers where the NMR is twice as high. A chi squared test showed that the association between wealth and neonatal mortality for both rural and urban dwellers was significant for those who had institutional deliveries: (p = <0.001 for rural and p = 0.02 for urban). For home deliveries the chi squared test was not significant for either urban or rural women.

**Table 2:** Descriptive analysis of mortality by place of birth and socio-economic grouping

**Wealth**	**Neonatal mortality rate (Confidence intervals in brackets)**	
**Wealth quintile 5 (richest)**	**Urban**	**Rural**
Home birth	26 (12–40)	34 (28–40)
Institutional birth	27 (16–39)	31 (16–45)
**Wealth quintile 1–4 (all but richest)**	**Urban**	**rural**
Home birth	36 (31–41)	42 (39–45)
Institutional birth	55 (41–69)	84 (61–107)
**Education**	**Neonatal mortality rate (Confidence intervals in brackets)**	
**No education**		
Home birth	44 (40–48)	
Institutional birth	101 (65–136)	
**Primary education**		
Home birth	37 (33–41)	
Institutional birth	82 (58–106)	
**Secondary education**		
Home birth	37 (32–43)	
Institutional birth	43 (32–55)	
**Higher education**		
Home birth	16 (5–27)	
Institutional birth	16 (7–25)	

**Figure 4:**
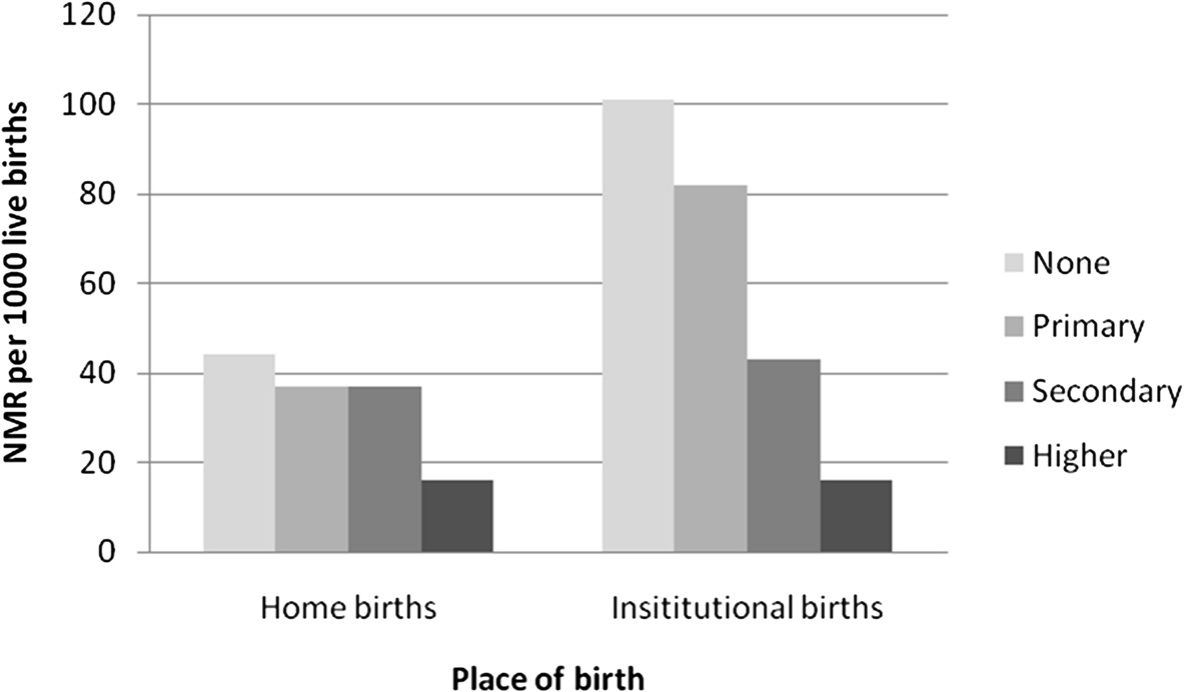
Neonatal mortality by mother’s education and place of birth.

Table 2 also shows differences for NMR by mother’s education and place of delivery. A similar pattern can be seen. Clearly babies whose mothers received no education or primary education had very markedly higher NMRs if born in institutions as compared with those born at home. Little difference was seen by place of birth among newborns whose mothers had secondary or further education. The graph in Figure 4 clearly demonstrates that differentials for mortality by level of mother’s education are much greater for babies born in government institutions than for those who were born at home. The chi squared tests are both significant but more so for the group giving birth in institutions: (p = <0.0001 for institutional births compared to a p =0.021 for home births).

As the period of time covered by the pooled surveys used for this study is quite long we also examined changes in NMR for institutional and home deliveries between the four surveys (see Table 3). Unfortunately small sample sizes made it impossible to further disaggregate by socio-economic class. While the pattern is not consistent, there is a more marked decline for those who gave birth in institutions than those who gave birth at home, and indeed a chi squared test was significant for institutional births, (p =0.036), but not for home births.

**Table 3:** Changes in neonatal mortality rate between surveys, disaggregated by institutional and home births

**Year of survey**	**NMR: Total sample**	**NMR: Institutional births only**	**NMR: Home deliveries only**
**1996/7**	45	72	43
**1999/2000**	42	49	41
**2004**	41	60	39
**2007**	38	37	37

### Results of multivariate analysis

The basic model was built on neonatal mortality as a dichotomous outcome. Six stratified models were developed:

•Model 1: Institutional delivery in urban areas, with urban wealth quintiles 1–4 and 5 as explanatory variables

•Model 2: Institutional delivery in rural areas, with rural wealth quintiles 1–4 and 5 as explanatory variables

•Model 3: Home delivery in urban areas, with urban wealth quintiles 1–4 and 5 as explanatory variables

•Model 4: Home delivery in rural areas, with rural wealth quintiles 1–4 and 5 as explanatory variables

•Model 5: Institutional delivery with mother’s highest level of education as explanatory variables

•Model 6: Home delivery with mother’s highest level of education as explanatory variables

When the models for institutional births are considered (Table 4), model 1 (rural only) shows a marked and significant increased OR of 2.25 (95% CI =1.18-4.28) for neonatal death for quintiles 1–4 (four poorest quintiles), whereas for model 2 (urban only) the OR of 1.7(95% CI = 0.79 -3.65) does not reach significance. For models 3 and 4 (home deliveries in rural and urban areas respectively) there is no increase in the OR for mortality for quintiles 1–4, suggesting wealth is not a significant factor. In model 5 (Table 5), using level of education as a candidate explanatory variable for institutional birth we find very large and significant increases in odds of neonatal death for women with no education (OR = 3.65, 95% CI = 1.47-9.07), and primary level education (OR = 3.07, 95% CI = 1.33-7.08) compared to the reference category of higher/further education. There is also a significant increased odds of mortality for babies of women who received secondary education (OR = 2.28, 95% CI = 1.06-4.91). When model 6 is considered (home deliveries) there are large increases in OR for women who received no, primary or secondary education, but they do not reach significance at the 5% level.

**Table 4:** **Regression models identifying factors that predict neonatal mortality among institutional and home births disaggregated by urban and rural samples with wealth quintiles as explanatory variable**^**b**^

**Odds ratios for neonatal mortality for institutional deliveries**								
**Variable**	**Model 1: Rural only**				**Model 2: Urban only**			
	**OR**	**P**	**Confidence intervals**		**OR**	**P**	**Confidence intervals**	
***Year of survey (reference 2007)***								
**2004**	1.41	0.41	0.62	3.21	1.12	0.78	0.52	2.40
**2000**	1.51	0.34	0.65	3.47	0.78	0.58	0.33	1.88
**1996**	2.70*	0.02	1.15	6.32	1.95	0.16	0.76	4.97
***Wealth quintile (reference richest quintile 5)***								
**Quintiles 1-4**	2.25**	0.01	1.18	4.28	1.70	0.17	0.79	3.65
	**Sample size: 1046**				**Sample size: 1733**			
**Odds ratios for neonatal mortality for home deliveries**								
	**Model 3: Rural**				**Model 4: Urban**				
**Variable**	**OR**	**P**	**Confidence intervals**		**OR**	**P**	**Confidence intervals**		
***Year of survey (reference 2007)***									
**2004**	0.93	0.59	0.70	1.22	1.25	0.37	0.76	2.06	
**2000**	0.88	0.36	0.67	1.16	1.57	0.07	0.97	2.55	
**1996**	1.61**	0.00	1.22	2.11	2.46**	0.00	1.36	4.45	
***Wealth quintile (reference richest quintile 5)***									
**Quintiles 1-4**	1.14	0.29	0.90	1.45	1.26	0.49	0.65	2.44	
	**Sample size: 18206**				**Sample size: 4943**				

* OR significant at 5% ** OR significant at 1%.

^b^ Models also adjusted for sex, multiple birth, birth order and parity and antenatal care.

**Table 5:** **Regression models to predict neonatal mortality for institutional and home births with mother’s education as explanatory variable**^**c**^

**Odds ratios for neonatal mortality for home and institutional deliveries**								
**Variable**	**Model 5: Institutional birth**				**Model 6: Home birth**			
	**OR**	**P**	**Confidence intervals**		**OR**	**P**	**Confidence intervals**	
***Year of survey (reference 2007)***								
**2004**	1.38	0.29	0.77	2.48	0.95	0.70	0.74	1.22
**2000**	1.09	0.79	0.59	2.01	0.93	0.54	0.72	1.18
**1996**	2.14*	0.03	1.10	4.16	1.63	0.00	1.26	2.10
**Maternal education (reference higher/further)**								
**No education**	3.65**	0.01	1.47	9.07	2.51	0.06	0.95	6.61
**Primary education**	3.07**	0.01	1.33	7.08	2.07	0.14	0.78	5.46
**Secondary education**	2.28*	0.04	1.06	4.91	2.11	0.13	0.79	5.61
**Sample size: 2803**					**Sample size: 23149**			

^c^ Models also adjusted for urban/rural, sex, multiple birth, birth order and parity and antenatal care.

## Discussion

### Institutional births and poor outcomes for the poor

The markedly larger socioeconomic differentials in neonatal mortality for those born in hospitals suggests strongly that provision of care at birth is particularly failing to meet the needs of disadvantaged women and their newborns. Both the descriptive and regression results clearly demonstrate that wealth has a markedly greater impact on neonatal outcomes for those giving birth in institutions compared to those giving birth at home. This suggests poor women are either arriving too late, or the care they receive is inadequate. While the descriptive analysis for education shows a very similar pattern, evidence from the multivariate analysis is less compelling: while not significant, there is a marked increase in OR for neonatal death for no education, primary and secondary education for women giving birth at home compared to those with higher education. The lack of significance may well be as a result of small sample size, and the findings may reflect the fact that the reference group of those women who have received further education is a particularly small and elite group with very low overall neonatal mortality.

The difference in neonatal outcomes from institutions between the urban and rural poor is also marked. This could be either because the characteristics of the two groups are different, or because access to services is more difficult in rural areas. In many countries rural poverty may be deeper and more chronic than in urban areas, and the population may be less educated [14]. There is some evidence that the rural population in this study are indeed different in character: we cannot directly compare wealth between urban and rural groups using the asset index, but if we examine education, over half (53%) of the rural quintiles 1–4 had no education compared to 34% of urban quintiles 1–4. However, another plausible reason could be the increased journey time when seeking care for rural women, which could detrimentally impact on outcome. The Bangladesh Maternal Health Services and Maternal Mortality Survey 2001 [8] found that only 26% of rural women with complications reported travelling less than 30 minutes to reach a provider or facility compared with 59% of urban respondents. Around 8% of women reported travelling for more than 3 hours, which obviously is likely to have negative consequences.

While utilisation data are often disaggregated, data on institutional outcomes are not routinely analysed – indeed the idea of inequity in outcomes from services is not well developed within the literature. This work suggests that such measures could be important, and provide a much more comprehensive picture than utilisation data alone. For instance, an apparent increase in utilisation among the poorest might mask the fact that many of these women are only arriving at hospital once complications are well advanced, thus reducing their chance of positive outcomes. Ideally such analysis should include attention to fresh stillbirths, which are often used as an indicator of care at birth. However, very limited data are available: DHS infrequently collect data on stillbirths, and quality is questionable, along with a further limitation that fresh and macerated stillbirths are not differentiated [15]. Indeed, it is worth considering that the extremely negative outcomes experienced by poor women would be even more concerning if stillbirths could be included, as poor or delayed intrapartum care may often result in the foetus dying during labour, so will not be included as a neonatal death.

The fact that due to sample size quintiles 1–4 were grouped together probably also results in an underestimation of the disadvantage faced by the poorest in terms of neonatal outcomes. Examining the difference in mortality between the top and bottom socio-economic groupings is a commonly used method of examining inequality, but has the limitation of failing to ascertain the distribution of mortality across the population. Another measure that could address this would be calculation of concentration curves or indices for the different groups as this takes into account mortality for the whole range of wealth. Further studies using this technique may be valuable.

Analysis over time of perinatal institutional outcomes can provide valuable evidence on quality of care. It would be assumed that as uptake of skilled care at birth increases, outcomes would improve as more and more women with uncomplicated pregnancies make an active choice to give birth in hospital. However, there are suggestions that in some cases increased demand actually has a negative impact on quality, as staff and resources are placed under greater pressure [16]. Monitoring of institutional mortality rates, particularly among the poorest could give some indication of any unintended negative consequences resulting from efforts to increase uptake of services.

#### Why are institutional outcomes so poor for women in Bangladesh?

While this study were able to identify groups of women who experienced particularly poor outcomes, our descriptive analysis is unable to demonstrate that any group has significantly reduced mortality as a result of utilising institutional care at birth when compared with babies born at home with similar socioeconomic characteristics. In an environment such as Bangladesh many mothers in the higher socio-economic groups may use hospital care at birth only in response to complications. While their outcomes may be better because of faster recognition of, and response to, complications than their poorer counterparts, their underlying condition will still affect the neonatal outcome.

It is also possible that if quality of care is relatively poor for all women (not just the poorest), the potential benefits from institutional birth may be reduced or in some cases even negated. There are ongoing concerns in connection with poor quality of maternal health care covering the entire period reflected in this study. An assessment of the Bangladesh Maternal Mortality programme in 1997 found patients’ wellbeing placed at risk by a lack of drugs and sterilised equipment and poor hygiene practices [17]. Dysfunctional staff configurations, inadequately skilled staff and poor infrastructure were also identified as barriers to quality care. More recently Chowdhury et al. [18] discovered constraints such as inadequate drug supplies, insufficient specialist staff, human resource absenteeism and long waiting times that all contributed to poor quality care. While data is again limited, there is also growing evidence that the potential for improved outcomes from an increase in facility births in developing countries may be reduced by the high incidence of hospital-acquired (nosocomial) infections in newborns [19].

As this study covers a considerable period of time, the question arises as to whether the situation has improved between 1997 and 2007. While Bangladesh has made some progress since the data used in this study was collected, the proportion of women receiving skilled care in Bangladesh is still amongst the lowest in the world and it could be questioned how much impact such limited progress would have on improving neonatal outcomes. Despite this, the limited data we present seems to suggest that most gains in reducing neonatal mortality have been in hospital births, and the most recent survey (2007) suggests that at the national level there are now similar levels of neonatal mortality in institutions and at home. This may imply that either quality has improved, or women with complications are recognising and seeking care more promptly. These changes can be considered within the rather surprising context of strong reductions in maternal mortality during this period. As the increase in skilled attendance has been so modest, it has been suggested that this reduction could be partly driven by improved knowledge of, and health seeking for complications, and increased access to emergency obstetric care, which would support our premise [20]. More research is needed to understand the factors driving reductions in maternal and neonatal mortality in Bangladesh, and how their impact can be maximised.

Efforts to improve timely uptake of services for all women (not just those with complications) is extremely challenging in contexts such as Bangladesh, and will require a number of both demand- and supply-side interventions to improve access to and quality of services and also increase understanding of the need for skilled attendance at all births. In addition, services must be accessible to all sectors of the community, which will require innovative and far-reaching solutions to solve the problems of both formal and informal health care costs.

## Conclusion

This study highlights that in Bangladesh babies from lower socio-economic groups have extremely poor outcomes if born in a facility. The mortality difference between richer and poorer, or educated versus uneducated is much more marked among facility births compared with the equivalent differential among home births, suggesting that services are particularly failing to meet the needs of poorer and less educated women. This could be because poor and uneducated women seek care later, or the care they receive is poorer quality.

Further research in different contexts (*e.g.* other countries with a higher proportion of facility births) may be able to identify improved outcomes for some groups of women. Greater disaggregation of institutional outcomes by socio-economic groups would provide more comprehensive evidence on whether services are reaching the poorest than utilisation data alone. As Bangladesh increases access to skilled attendance further analysis could provide an important insight into the country’s success in providing pro-poor care. Indeed, as countries move nearer to the Millennium Development Goals the focus should shift from overall mortality rates to the key differences between mortality rates for different socioeconomic groups. As institutionalisation of births increases, more focus should be placed on ensuring services promote rather than reduce equity of outcomes.

## Endnotes

^a^It was also noted that the asset scores for quintiles 1–4 in both urban and rural areas showed little real difference in assets between these poorest four quintiles.

## Competing interests

The authors declare no competing interest in relation to the article.

## Authors’ contributions

SN planned the study and performed the statistical analyses with advice from ZM. SN prepared the first draft of the manuscript. ZM commented extensively on the first draft and edited the final manuscript. Both authors read and approved the final manuscript.
